# Reversible Histone Modifications Contribute to the Frozen and Thawed Recovery States of Wood Frog Brains

**DOI:** 10.3390/biom14070839

**Published:** 2024-07-12

**Authors:** Tighe Bloskie, Olawale O. Taiwo, Kenneth B. Storey

**Affiliations:** Institute of Biochemistry and Department of Biology, Carleton University, 1125 Colonel By Drive, Ottawa, ON K1S 5B6, Canada; tighebloskie@cmail.carleton.ca (T.B.); olawaletaiwo@cmail.carleton.ca (O.O.T.)

**Keywords:** epigenetics, histone modifications, freeze tolerance, hypometabolism, wood frog, brains

## Abstract

Epigenetic regulation, notably histone post-translational modification (PTM), has emerged as a major transcriptional control of gene expression during cellular stress adaptation. In the present study, we use an acid extraction method to isolate total histone protein and investigate dynamic changes in 23 well-characterized histone methylations/acetylations in the brains of wood frogs subject to 24-h freezing and subsequent 8-h thawed recovery conditions. Our results identify four histone PTMs (H2BK5ac, H3K14ac, H3K4me3, H3K9me2) and three histone proteins (H1.0, H2B, H4) that were significantly (*p* < 0.05) responsive to freeze-thaw in freeze-tolerant *R. sylvatica* brains. Two other permissive modifications (H3R8me2a, H3K9ac) also trended downwards following freezing stress. Together, these data are strongly supportive of the proposed global transcriptional states of hypometabolic freeze tolerance and rebounded thawed recovery. Our findings shed light on the intricate interplay between epigenetic regulation, gene transcription and energy metabolism in wood frogs’ adaptive response to freezing stress.

## 1. Introduction

Epigenetics refers to functional changes to gene activity without modification of encoded DNA sequences. Epigenetic control is stable, dynamic and responds to various environmental stimuli. Alongside transcription factors, epigenetic regulation plays a ubiquitous role in transcriptional control of gene expression, exerting a major influence on the transcriptome of cells. Aberrant and dysregulated epigenetic mechanisms are, therefore, unsurprisingly implicated in the development and maintenance of all types of human malignancies [1]. These reversible modifications play a fundamental role in regulating various biological processes, including development, cellular differentiation, and response to environmental cues [2,3]. Recently, epigenetic regulation has emerged as a critical control of transcriptional and translational processes in support of hypometabolic stress adaptation [4]. This is becoming increasingly evident in wood frogs (*Rana sylvatica*), who possess a unique ability to accept upwards of 70% of their body water as extracellular ice in an overwintering phenomenon known as freeze tolerance [5,6].

During freeze tolerance, wood frogs undergo a series of physiological and biochemical changes to protect their tissues from damage caused by ice crystal formation. They supercool their body fluids slightly (2–3 °C) and use ice-nucleating proteins (INPs) to allow for slow, controlled ice formation within extracellular and extra-organ spaces. Glycogenolysis of their large hepatic glycogen stores supplies immense concentrations (200–300 mM) of glucose that is utilized as a colligative cryoprotectant of intracellular environments. Various cytoprotective processes, like chaperone proteins and antioxidant defenses, are upregulated to preserve essential biological macromolecules [7]. The principal component of wood frog freeze survival is metabolic rate depression (MRD), where hibernating anurans drastically reduce their energetic demand to support the limited carbohydrate stores as a result of the anoxic and non-feeding natures of the months-long dormant period [8]. The synthesis of nascent proteins is among the most intensive subcellular processes consuming roughly 5 adenosine triphosphate (ATP) equivalents per peptide bond and 25–40% of the energetic allowance [9,10]. It is therefore unsurprising that suppression (>90%) of protein translation is a core feature of animal MRD [10,11,12], mediated through the collective action of (1) transcriptional, (2) post-transcriptional and (3) translational controls.

Transcriptional gene regulation is heavily implicated in wood frog freeze tolerance as mRNA synthesis is also a significant contributor to ATP usage and hypometabolism necessitates the induction of various pro-survival genes, including Fr10, li16 and fibrinogen [13,14,15]. Evidence for tissue-specific reversible epigenetic regulation of wood frog freeze tolerance and thawed recovery continues to amass in recent years, particularly via histone post-translational modifications (PTMs). In hepatic studies, hypomethylation of permissive H3K4me1, linked to reductions of putative methyltransferase (MTase) SMYD2 and MLL complex-associated protein ASH2L may contribute to transcriptional silencing during wood frog freezing [16], while permissive methyl-arginine marks H3R17me2a and H4R3me2a, correlated with heightened PRMT1/4 expression/activity may play a role in facilitating rebounded transcriptional activities upon thawing [17]. Support for histone PTM-mediated transcriptional activation during wood frog thawed recovery is similarly found via studies on the brain, which detail coordinated reductions in repressive H3K9me3, MTases SUV39H1/ESET and H3K9 MTase activity [6].

These preliminary investigations indicate that histone PTMs play a critical role in synchronizing freeze tolerant and thawed recovery adaptive responses, providing rapid, reversible transcriptional control during consequential cellular stresses like freezing, anoxia and reperfusion. Histone modifications, chiefly lysine methylation/acetylation and arginine methylation control gene expression and chromatin structure [18]. Lysine residues of core histone proteins are commonly acetylated by lysine acetyltransferases (KATs) and reversed by histone deacetylases (HDACs) and sirtuins (SIRTs)—facilitating sites for active local gene transcription through attenuated histone-DNA ionic interactions and permissive acetyl-binding bromodomain (BRD) complexes. Alternatively, lysine and arginine residues are methylated by lysine MTases (KMTs) and protein arginine MTases (PRMTs), respectively, and removed largely by Jumonji-C domain lysine demethylases (KDMs). Transcriptional regulatory influence of histone methylation is substrate-specific —various chromatin immunoprecipitation (ChIP) approaches have mapped some methyl-histone marks around transcription start sites (TSSs) of active genes (i.e., H3K4me2/3, H3K36me3, H3R17me2a), and others with silenced genes (i.e., H3K9me2, H3K27me3, H4R3me2s) [19,20,21]. Additionally, the cofactor dependence of various histone-modifying enzymes (acetyl-CoA for KATs, NAD^+^ for SIRTs, S-adenosylmethionine (SAM) for MTases, O_2_ for KDMs) highlights not only a tight interplay between histone lysine acetylation with metabolically reprogrammed states [22] but also histone methylation/demethylation with oxygen-limiting subcellular environments [23]; facets that are both relevant to wood frog freeze tolerance. With the growing list of histone PTMs (>100) and the elucidation of their functions, there is an obvious need for comprehensive profiling approaches for studies on animal extreme stress adaptation. 

In the current study, we use an acid extraction/trichloroacetic acid precipitation method to purify total histone protein [24] from wood frog brain tissue and subsequently examine the global levels of 23 well-characterized histone PTMs across the freeze-thaw cycle (24-h freezing, 8-h thawed recovery). Through probing the functional consequences of altered histone modifications, we aimed to understand how changes in histone PTMs in frozen wood frog brains influence gene expression, chromatin structure, and potential physiological adaptations related to freeze tolerance. Through this research, we provide evidence for the role of epigenetic mechanisms in freeze tolerance with the aim of contributing to the knowledge gap of epigenetic regulation in the context of freeze tolerance, specifically focusing on histone changes as potential mechanisms that facilitate adaptive responses to freezing stress.

## 2. Materials and Methods

### 2.1. Animal Experiments

Male adult wood frogs (*Rana sylvatica*) weighing 5–7 g were captured from breeding ponds near Ottawa, Ontario, during the early spring season. The frogs underwent a tetracycline bath and were housed in plastic containers containing damp sphagnum moss. After a period of acclimation (with no feeding) at 5 °C for two weeks, control frogs were selected from this group for sampling. To initiate the freezing treatment, a subset of the remaining frogs was transferred to plastic containers lined with damp paper towels. These containers were then placed in an incubator set to −4.0 °C for 45 min, facilitating ice nucleation on the skin through contact with forming ice crystals in the paper. The incubation temperature was subsequently adjusted to −2.5 °C and maintained for 24 h. This treatment protocol ensured that approximately 65–70% of the total body water froze as extracellular ice. Half of the frogs were sampled while in a frozen state, while the remaining frogs were returned to a 5 °C incubator for 8 h, allowing them to fully thaw before sampling took place. The experimental state of freezing is verified by calorimetry and percent ice determinations well-described in the literature [25]. A thermocouple placed against the surface of the frog can identify the spike in body temperature caused by ice formation and monitor it until thermal equilibrium and beyond. Characteristics of frozen frogs are stiff/brittle limbs, hard abdomens and cloudy eyes. Large ice crystals are observed in the leg muscles and extra-organ abdominal cavity. Ice surrounds the organs, but they are not frozen while breathing/heartbeat are undetected. Frozen frogs are placed in insulated water containers at 20 °C and allowed to thaw. The cooling of surrounding water as compared to pure ice and water of the same size at −4 °C can estimate the percent body weight as ice. Thawing is evident by resumed heartbeat, breathing and behavioral/locomotor activities by 8 h. Control, 24 h frozen, and 8 h thawed frogs were euthanized by pithing and then brain tissues were excised and immediately frozen in liquid nitrogen. Tissues were stored at −80 °C until used. All protocols were approved by the Carleton University Animal Care Committee within the guidelines of the Canadian Council on Animal Care.

### 2.2. Total Histone Extraction—Modified from [24]

Roughly 80 mg of harvested wood frog brain tissue (n = 5 control, 24 h frozen and 8 h thawed) was pulverized into small pieces under liquid nitrogen using mortar and pestle. Biological replicates from each experimental treatment were pools of three to four different *R. sylvatica* brains. Frozen tissue pieces were transferred to a glass-glass Dounce and homogenized via piston strokes in 4 volumes of Triton Extraction Buffer (TEB: PBS containing 0.5% Triton X-100 (*v*/*v*), 0.02% (*w*/*v*) NaN_3_, 10 uL/mL protease inhibitor cocktail (BioShop, Cat# PIC001.1, Burlington, ON, Canada), 1 mM sodium orthovanadate, 10 mM β-glycerophosphate) to disaggregate tissue. Homogenates were incubated on ice for 30 min with occasional vortexing before spinning samples at 10,000× *g* for 10 min at 4 °C to pellet nuclei. Non-nuclear supernatants were discarded, and nuclear pellets were washed in half the volume of TEB and centrifuged as previously (10,000× *g* for 10 min at 4 °C). Again, supernatants were removed. Nuclei were then acid-extracted in 400 μL 0.2 M H_2_SO_4_ on a rotator at 4 °C for 1 h. Histones are highly basic proteins that solubilize into the supernatant, while most other nuclear proteins are left behind in the debris. Samples were centrifuged at 12,000× *g* for 10 min at 4 °C to pellet nuclear debris. Histone supernatants were transferred to fresh tubes and precipitated in trichloroacetic acid (TCA). A total of 132 μL of 100% TCA (500 g in 227 mL ddH_2_O) was dropped and solutions were incubated on ice for 1 h. Samples turned cloudy over time. Histones were pelleted at 12,000× *g* for 10 min at 4 °C (appearing as a cloud up the wall of tube) and washed in ice-cold acetone carefully to remove residual acid. After a final spin at 12,000× *g* for 10 min at 4 °C, acetone was removed and histone pellets were air-dried at room temperature for 20 min. Histone proteins were resuspended in 100 μL of autoclaved ddH_2_O and sonicated to help with solubility.

Histone concentrations were quantified by the BioRad protein assay (BioRad Laboratories, Cat# 5000002, Hercules, CA, USA) and standardized to 0.5 μg/μL in 2X sodium dodecyl sulfate (SDS) buffer (100 mM Tris-base, 4% *w*/*v* SDS, 20% *v*/*v* glycerol, 0.2% *w*/*v* bromophenol blue, 10% *v*/*v* 2-mercaptoethanol). A total of 1 μg of histone extracts were visualized by staining with Coomassie (0.25% *w*/*v* Coomassie brilliant blue, 7.5% *v*/*v* acetic acid, 50% *v*/*v* methanol) following 15% SDS-PAGE. 

### 2.3. Western Immunoblotting

A total of 1–4 μg of equal total histone content was loaded into discontinuous SDS-polyacrylamide gels with 4 μL of BLUelf Prestained Protein Ladder (10–245 kDa; FroggaBio, Cat# PM008-0500, Concord, ON, Canada) molecular weight standards. Separating gels were 15% acrylamide:bis-acrylamide *v*/*v* in 1.5 M Tris buffer (pH 8.8), 0.1% SDS, 0.1% ammonium persulfate (APS), and 0.1% N,N,N′,N′-tetramethylethylenediamine (TEMED) while stacking gels contained 5% acrylamide:bis-acrylaminde *v*/*v* in 1 M Tris buffer (pH 6.8), 0.1% SDS, 0.1% APS and 0.1% TEMED. Purified histones were electrophoresed at 150-180V for roughly 60 min using the BioRad Mini Protean III system (BioRad Laboratories, Hercules, CA, USA) in 1X Tris-glycine running buffer (25 mM Tris-base, 190 mM glycine, 0.1% *w*/*v* SDS, pH 8.3–8.5). Size-separated histones were then wet electroblotted onto 0.45 μm polyvinylidene difluoride (PVDF) membranes at ~50 V for 45 min in 1X Tris-glycine transfer buffer (25 mM Tris-base, 190 mM glycine, 20% *v*/*v* methanol, pH 8.3–8.5). Post-transfer, histone membranes were blocked in 5% *w*/*v* non-fat dried skim milk in 1X Tris-buffered saline (20 mM Tris-base, 140 mM NaCl, pH 7.5) with 0.05% Tween-20 (TBST) via rocking for 30 min at room temperature. Membranes were washed 3 times in 1X TBST for 5 min to remove excess milk before overnight (roughly 18 h) 4 °C probing with histone PTM-specific anti-rabbit primary antibodies (1:1000 *v*/*v* in 1X TBST, 0.02% *w*/*v* sodium azide).

All primary antibodies in the present study are from commercially available suppliers including: (1) ABclonal Technology (Histone H1.0, A3298; Histone H3, A2348; Histone H4, A17024; H3K4me2, A2356; H3K4me3, A2357; H3K9me2, A2359; H3K27me3, A2363; H3K36me3, A2366; H3K79me3, A2369; H4K20me3, A2372; H2AK5ac, A15620; H2BK5ac, A15621; H3K14ac, A7254; H3K18ac, A7257; H3K27ac, A7253; H3R2me2s, A2373; H3R17me2a, A2421; H3R26me2a, A2375; H4R3me2a, A2376; Woburn, MA, USA), (2) Cell Signaling Technology (Histone H2A, #2578P; Histone H2B, #8135P; H3K9ac, #9649P; H3K23ac, #9674S; H4K8ac, #2594P; Danvers, MA, USA), (3) MyBioSource (H3R2me2a, MBS9402172; H3R8me2s, MBS9607605; H4R3me2s, MBS126222; San Diego, CA, USA) and (4) Abbexa Ltd. (H3R8me2a, abx000031; Cambridge, UK).

Membranes were washed in 1X TBST for 3 × 5 min the following day before 30 min room temperature rocking incubations in horseradish peroxidase-linked goat anti-rabbit secondary antibodies [BioShop, Cat# APA007P.2, Burlington, ON, Canada) (1:8000–10,000 *v*/*v* in 1X TBST)]. Western immunoblot bands were visualized by enhanced chemiluminescence (ECL) with substrates H_2_O_2_ and Luminol using a ChemiGenius Bio Imaging System (Syngene, Frederick, MD, USA) following final 3 × 5 min washes in 1X TBST. Whole histone immunoblots are shown in Figure S1. For loading control, ECL band intensities were standardized against Coomassie-stained total core histone content (11–17 kDa) from the same lane.

### 2.4. Quantification and Statistics

Imaged ECL band intensities were quantified by densitometry using GeneTools Software (version 4.3.8.0, Syngene, Frederick, MD, USA). Relative ECL values were standardized against Coomassie-stained total core histone content (11–17 kDa) for protein loading control. Histograms show mean values ± SEM for n = 4–5 biological replicates from different pools of control, 24-h frozen and 8-h thawed wood frog brains. Statistical significance testing was completed by one-way analysis of variance (ANOVA) and post-hoc Tukey’s tests (*p* < 0.05) using SigmaPlot 12.0 statistical software (Systat Software Inc., San Jose, CA, USA).

## 3. Results

### 3.1. Histone H1.0, H2B and H4 Are Responsive to the Wood Frog Brain Freeze-Thaw Cycle

**Figure 1:**
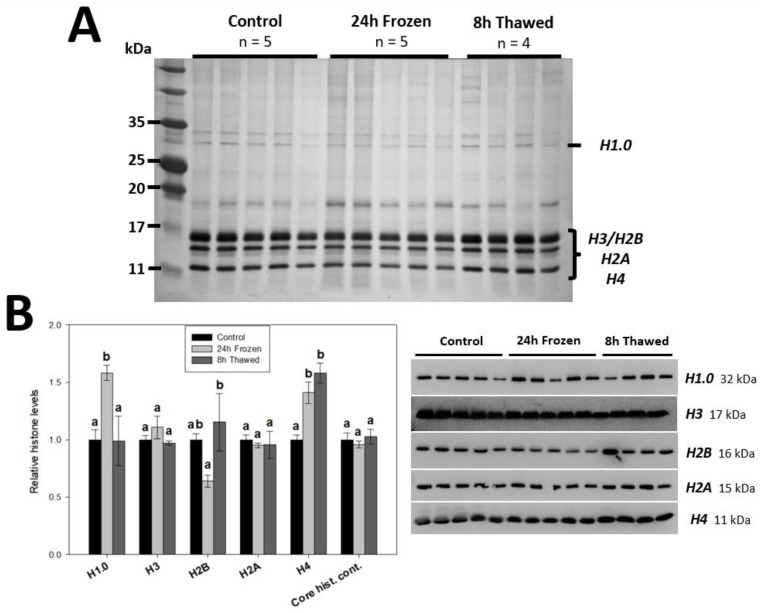
(**A**) A total of 1 μg of total histone extracts from *R. sylvatica* brain across three treatments (control, 24-h frozen, and 8-h thawed) using 15% SDS-PAGE. The four core histone bands are clearly visible in the histone range (11–17 kDa) following Coomassie staining. (**B**) Relative expression of individual histones, from largest to smallest (H1.0, H3, H2B, H2A and H4), across the freeze-thaw cycle of *R. sylvatica* brains as determined by Western immunoblotting. Histograms show mean values ± SEM for n = 4–5 biological replicates of purified histones from control, 24-h frozen and 8-h thawed wood frog brains. Coomassie-stained core histone content is included. Data were analyzed using one-way analysis of variance (ANOVA) with post-hoc Tukey’s tests (*p* < 0.05). Values denoted by the same letter are not significantly different from each other.

Total histone extracts from control, 24 h frozen and 8 h thawed *R. sylvatica* brains are shown in Figure 1A. The presence of four distinct core histone bands (H3, H2B, H2A, H4) in the histone range (11–17 kDa) as well as linker histone H1.0 was verified by immunoblot analysis. Interestingly, significant (*p* < 0.05) changes were observed in the expression levels of H1.0, H2B, and H4 (Figure 1B). H1.0, a linker histone, displayed upregulated expression 1.58 ± 0.06-fold after 24 h freezing relative to controls, indicating a potential role in response to freezing stress, before returning to control values during thawed recovery. Core histones H4 and H2B were also responsive to the freeze-thaw cycle. H4 levels were similarly elevated 1.41 ± 0.09-fold under frozen conditions, however, remained high (1.58 ± 0.09-fold) upon thawing, while there was a significant (*p* < 0.05) increase in H2B expression during the transition from frozen to thawed states (control vs. frozen; *p* = 0.149). H3 and H2A levels were consistent across all experimental conditions, suggesting their stable expression in response to wood frog freezing and thawing.

### 3.2. H2BK5ac and H3K14ac Are Reduced across Chromatin of Frozen Wood Frog Brains

**Figure 2:**
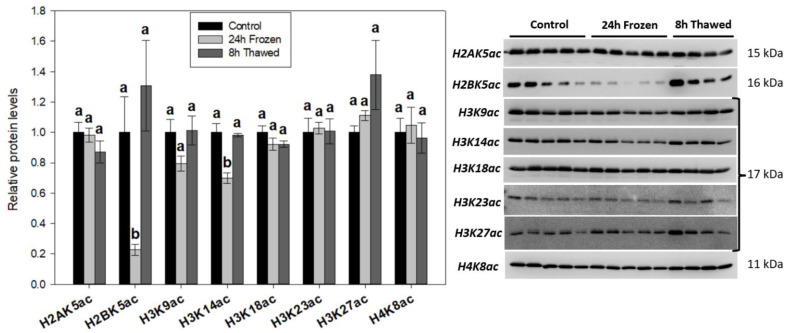
Relative abundance of prominent histone acetyl-lysine modifications during the freeze-thaw cycle of *R. sylvatica* brains as determined by Western immunoblotting. Histograms show mean values ± SEM for n = 4–5 biological replicates of purified histones from control, 24-h frozen and 8-h thawed wood frog brains. Data were analyzed using one-way analysis of variance (ANOVA) with post-hoc Tukey’s tests (*p* < 0.05). Values denoted by the same letter are not significantly different from each other.

Western immunoblotting of total histone extracts was used to assess the relative expression of eight well-characterized histone acetyl-lysine modifications (H2AK5ac, H2BK5ac, H3K9ac, H3K14ac, H3K18ac, H3K23ac, H3K27ac, H4K8ac) in wood frog brains across three experimental conditions: control, 24 h frozen, and 8 h thawed recovery (Figure 2). H2BK5ac abundance was strongly depressed (*p* < 0.05) under frozen conditions (to 23 ± 3% of controls), before rebounding to normothermic levels during recovery. Depleted H2BK5ac can only be partially explained by changes in histone H2B (Figure 1B), as it is still at the significance threshold (*p* = 0.050) when standardized to total H2B. H3K14ac was another freeze-responsive acetyl-lysine mark—reduced to 70 ± 3% of controls during freezing stress. No significant changes were observed across the other acetyl-lysine PTMs, though it is noteworthy that H3K9ac (*p* = 0.124) followed a similar trend with H3K14ac. 

### 3.3. Permissive H3K4me3 and Repressive H3K9me2 Display Opposing Trends during Freeze-Thaw

**Figure 3:**
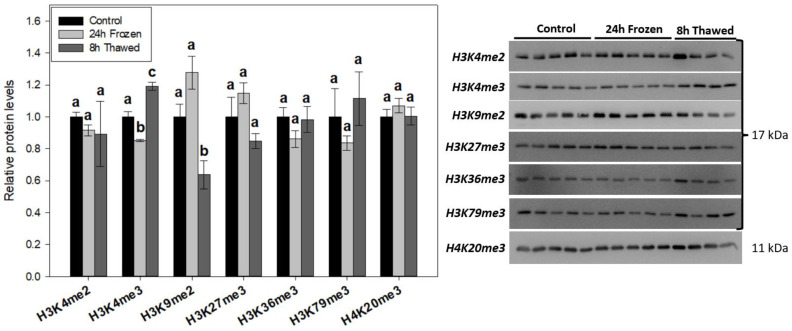
Relative abundance of prominent histone methyl-lysine modifications during the freeze-thaw cycle of *R. sylvatica* brains as determined by Western immunoblotting. Histograms show mean values ± SEM for n = 4–5 biological replicates of purified histones from control, 24-h frozen and 8-h thawed wood frog brains. Data were analyzed using one-way analysis of variance (ANOVA) with post-hoc Tukey’s tests (*p* < 0.05). Values denoted by the same letter are not significantly different from each other.

The relative levels of seven histone methyl-lysine marks (H3K4me2, H3K4me3, H3K9me2, H3K27me3, H3K36me3, H3K79me3, H4K20me3) were similarly investigated via immunoblotting of *R. sylvatica* brain total histone extracts across the freeze-thaw cycle (Figure 3). H3K4me3, a mark tightly linked to transcriptional activation, displayed levels that correlate well with the presumed transcriptional state of hypometabolic freeze tolerance and hypermetabolic freeze recovery. H3K4me3 abundance dropped to 85 ± 1% of controls in the frozen state followed by thaw-mediated induction (1.19 ± 0.02-fold vs controls) during recovery. Biological variation was very tight for this epigenetic mark, making all experimental groups statistically significant (*p* < 0.05) from one another. H3K9me2, a repressive histone PTM, was conversely attenuated to 64 ± 9% of controls during 8 h thawing, levels that were significant from both other treatments. Frozen H3K9me2 expression trended upwards (*p* = 0.109) relative to controls, while levels of another repressive mark, H3K27me3, trended downwards (*p* = 0.109) after 8 h thawing relative to the frozen condition. No significant changes were observed across any of the other studied methyl-lysine histone PTMs.

### 3.4. Freeze-Thaw Led to No Significant Changes in Histone Methyl-Arginine Modifications

**Figure 4:**
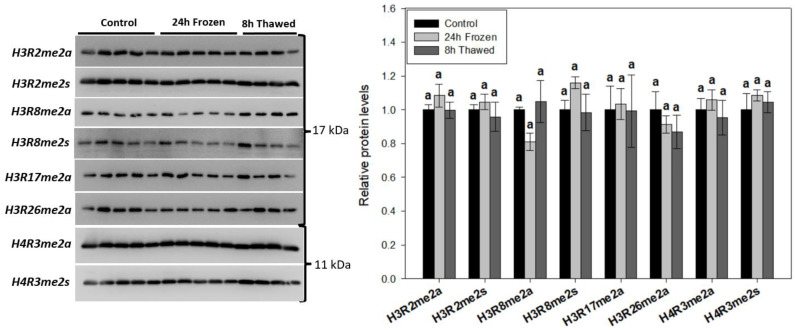
Relative abundance of prominent histone methyl-arginine modifications during the freeze-thaw cycle of *R. sylvatica* brains as determined by Western immunoblotting. Histograms show mean values ± SEM for n = 4–5 biological replicates of purified histones from control, 24-h frozen and 8-h thawed wood frog brains. Data were analyzed using one-way analysis of variance (ANOVA) with post-hoc Tukey’s tests (*p* < 0.05). Values denoted by the same letter are not significantly different from each other.

Eight histone methyl-arginine PTMs (H3R2me2a, H3R2me2s, H3R8me2a, H3R8me2s, H3R17me2a, H3R26me2a, H4R3me2a, H4R3me2s) were tracked during freeze-thaw via immunoblotting of wood frog brain-extracted total histone protein (Figure 4), same as above. Our data reveal no statistically significant (*p* < 0.05) changes in any methyl-arginine marks. It is noteworthy that H3R8me2a, a permissive histone PTM, trended downwards during freezing and back upwards during 8 h recovery (*p* = 0.076). 

## 4. Discussion

Histone PTMs play a critical role in regulating gene expression and modulating chromatin structures. They have been heavily implicated in various biological processes, including development, cellular differentiation, disease states and response to environmental stimuli [1,2,3]. However, much less is known about the roles of histone dynamics under ectothermic extreme stress adaptation to conditions such as whole-body freezing and subsequent thawing. In this study, we aimed to investigate if changes in histone modifications contributed to the frozen state of wood frog brains—anurans well-known for their remarkable freeze tolerance ability. We modified the existing total histone extraction protocol to isolate histone protein from frozen tissue [24] and used Western immunoblotting techniques to quantify the relative levels of specific histone proteins and notable PTMs. This comprehensive analysis of the 5 major histone proteins and 23 putative methylations/acetylations is among the first of its kind, seeking to elucidate the potential impact of freezing and thawing conditions on histone dynamics and its implications for transcriptional gene regulation in wood frog brains.

### 4.1. Freeze-Thaw Stimulates Dynamic Changes of Some Histone Proteins in Wood Frog Brain Chromatin

The differential expression patterns of histones H1.0, H2B, and H4 across wood frog brain freeze-thaw (Figure 1B) provide intriguing insights into the potential rewiring of chromatin structure and gene transcription in response to extreme environmental conditions. Histone H1.0 is a conserved subtype of H1 that binds to core histone octamers to complete nucleosome structures. Interestingly, overexpression of H1.0 leads to reduced transcript levels of all RNA polymerase II genes in mouse fibroblasts [26] and strongly inhibits DNA replication in Xenopus nuclei [27], effects not seen in other H1 subtypes. The 1.58 ± 0.06-fold enrichment of H1.0 in total histone extracts following 24-h freezing suggests it contributes to the repressed transcriptional and arrested cell cycle states of hypometabolic freeze tolerance. Histone H2B is a core histone component of the nucleosome that carries key acetylated modifications like H2BK5ac. The thaw-responsive (*p* < 0.05) induction of H2B protein levels, relative to freezing conditions, correlated well with H2BK5ac (Figure 2) and may act as a means to control this PTM. H2BK5ac was the most responsive modification in brains during freeze-thaw, displaying a >4-fold reduction in abundance under frozen conditions, which were returned to control levels after thawing for 8 h. H2BK5ac is a dynamic marker of active gene promoters, and a recent study highlights that the presence/absence of H2BK5ac confidently predicts the transcriptional activity of more than 75% of all genes in human CD4+ T-lymphocytes [28]. Whether other H2B PTMs correlate with H2B and H2BK5ac across wood frog freeze-thaw is a topic of future study. 

The upregulation of histone H4, 1.41 ± 0.09-fold after 24 h of freezing and 1.58 ± 0.09-fold following the 8-h thawed period indicates its potential involvement in the freeze tolerance mechanism. Histone H4 is another core histone protein commonly modified to control gene transcription. Somewhat surprisingly, H4 levels did not correlate with any tested modifications (H4K20me3, Figure 2; H4K8ac, Figure 3; H4R3me2a/s, Figure 4), suggesting an independent mechanism. Numerous studies have identified major antimicrobial roles for H4 and H4-derived peptides as part of the innate system of multiple animals, including humans [29]. In particular, one study has shown that H4 expression is induced in the hemolymph of American cupped oysters, *Crassostrea virginica*, playing a key antibacterial role in response to infection [30]. It is, therefore, possible that elevated levels of H4 could contribute to the brain immunological response of wood frogs during the freeze-thaw period. Immune suppression is an unfortunate risk factor for hibernating animals, particularly evident in the white nose syndrome of bats [31]. Heightened H4 expression across freeze-thaw may serve a protective role in the brains of these vulnerable anurans during prolonged freeze tolerance. A recent study has shown all histone H4 genes, but not the genes of other core histones (like H2A), are regulated by Akt and NF-κB signaling pathways in mammalian A549 and H2009 cells [32]. It is, therefore possible, that Akt and/or NF-κB activation could specifically stimulate histone H4 expression across freeze-thaw. Interestingly, cellular markers consistent with both Akt and NF-κB activation have been observed across the freeze-thaw cycle of wood frog liver [33,34], but are understudied in the brain. Core histones H3 and H2A were unaffected by freezing or thawing conditions (Figure 1B). Interestingly, heightened H4 expression but not H3 under frozen conditions was similarly observed in the freeze-tolerant gall fly, *Eurosta solidaginis* [35].

Regulation of histone protein expression, alongside the growing characterization of histone variants, has emerged as a critical control of gene expression [36]. In one study, by knocking down stem-loop binding protein (SLBP), researchers show modest decreases in histone H2B, H2A.X and H3. Consequently, they observed an accelerated RNA polymerase II elongation rate, due to the de-repression of the nucleosomal barrier effect, which in turn disrupted the proper temporal coupling of transcription and splicing, and led to subtle expression changes of many genes [37]. In another paper, scientists generated mammalian *hmgb*1−/− cells which reduced histone content by 20–30%. As a result, these cells contained fewer nucleosomes, coinciding with higher global mRNA content from increased transcription of ~10% of total genes [38]. They saw a similar effect with yeast *nhp6* mutants. The freeze-thaw responsive (*p* < 0.05) nature of histone H1.0, H2B and H4 protein expression in wood frog brains may play a key undiscovered role in the transcriptional control of freeze tolerance.

### 4.2. Histone H3 PTMs Provide Evidence in Favour of Silenced Gene Transcription under Frozen Conditions

The brains of *R. sylvatica* exhibited some key differences in histone H3 PTMs after frogs were frozen for 24 h. Notably, freezing stress triggered significant (*p* < 0.05) reductions in key permissive modifications, H3K4me3 (to 85 ± 1% of controls; Figure 3) and H3K14ac (to 70 ± 3% of controls; Figure 2). These correlated with downward trends of other active histone marks H3R8me2a (Figure 4) and H3K9ac (Figure 3), while an upward trend in repressive H3K9me2 levels (Figure 3) was also observed in the brains of frozen anurans. Collectively, histone H3 dynamics agree with previous literature on animal hypometabolism [10,11,12], and provide strong evidence in favor of suppressed global gene transcription and likely subsequent protein translation to support the hypometabolic demands of freeze tolerance. 

Lessened brain H3K4me3 levels after 24-h freezing suggest a potential repressive role on transcription in the frozen state, possibly impacting gene expression programs of non-essential pathways. H3K4me3 has a well-documented association with active gene promoters [19], via the activity of the evolutionarily-conserved KMT2 family of methyltransferases [39]. Interestingly, in mouse embryonic stem cells (mESCs), H3K4me3 is more rapidly lost than H3K4me1/2 by KDM5A/B demethylases [40], which might explain why H3K4me2 and H3K4me1 were statistically unchanged as we see here (Figure 3) and previously [6]. This study also linked depleted H3K4me3 to slowed RNA Pol II elongation and lessened global RNA output. Lowered H3K4 methylation has similarly been documented in hepatic studies of frog freeze tolerance and turtle anoxia tolerance [16,41]. Studies into the roles of lysine demethylases, notably KDM5 members, are ongoing.

H3K9ac and H3K14ac co-exist genome-wide as part of the active promoter state of regulatory elements in mouse mESCs [42]. They also correlate strongly with H3K4me3, which is highly agreeable with our results here. The significant reductions of H3K4me3 and H3K14ac as well as the downward trend of H3K9ac in frozen brains point to a coordinated mechanism of action. In another hypometabolic system, the reduction in these three modifications at promoters is integral to the torpor-mediated knockdown of hibernating proteins, particularly HP25, in Siberian chipmunks (*Tamias asiaticus*) [43]. Attenuated H3K14ac levels are observed as part of *E. solidaginis* responses to freezing and red-eared slider (*Trachemys scripta elegans*) responses to anoxia [35,44], coinciding with our data. Future studies with larger sample sizes may elucidate the significance of H3K9ac in the freeze-thaw response, as H3K9ac depletion via heightened HDAC expression has been suggested in anoxic *T.s. elegans* [45]. 

Our final observations are the opposing trends of repressive H3K9me2 and adjacent permissive H3R8me2a under frozen conditions, both of which are consistent with the proposed suppressed transcriptional state. Elevated H3K9me2 in frozen wood frog brains, which may be identified by further study with larger sample sizes, could be due to activation of the hypoxia-inducible methyltransferase G9a [46], as oxygen limitation is a prominent subcellular byproduct of freezing. Elevated G9a expression/activity is notable in hypometabolic anoxia tolerance of freshwater turtles [41], while frozen wood frog brain H3K9me2 levels correlate with those in anoxic skeletal muscles [47]. Interestingly, H3R8me2a has been shown to antagonize G9a-mediated H3K9me2 in vitro [48], which coincides with their opposing trends across freeze-thaw. Future work could elucidate the potential role of H3R8me2a/H3K9me2 crosstalk and their contribution to transcriptional regulation in wood frog brain freeze tolerance.

### 4.3. Rebounded Transcriptional State during Wood Frog Brain Thawing?

Freezing and subsequent thawing can cause cellular damage, including DNA and cytoskeletal damage [5]. Thawing is also particularly consequential because of the rapid reperfusion and increased ROS generation from reactivated electron transport chains. The restoration of normal cellular functions, the implementation of damage repair pathways and the maintenance of cell protective mechanisms require the resumption of normothermic transcriptional networks. Evidence for transcriptional rebounding has been observed previously in wood frog thawed recovery [6,49] but also in early arousal states of hibernating ground squirrels [50,51]. 

Key thaw-responsive (*p* < 0.05) histone PTMs in the brains of wood frogs include permissive H3K4me3 and repressive H3K9me2 (Figure 3). Global H3K4me3 levels were elevated 1.19 ± 0.02-fold while H3K9me2 abundance dropped to 64 ± 9% of control brains. Also noteworthy is the downward trend of H3K27me3, another repressive histone mark, as well as returned H2BK5ac/H3K14ac abundance. These data contrast strongly with frozen samples and are indicative of recovered gene transcription activity in agreement with previous literature. The demand for transcriptional activation at 8 h post-freezing may even be above the normoxic level, as a brief “overshoot” of O_2_ uptake and CO_2_ production is observed around this stage of thawing [8]. This overshoot likely reflects the need to address accumulated anaerobic byproducts, as well as macromolecular and tissue damage caused by the freeze-thaw event. The exact mechanisms linking elevated H3K4me3 to the relaxed chromatin and activated transcriptional states during thawed recovery in frozen wood frog brains require further investigation.

H3K9me2 abundance was significantly (*p* < 0.05) depressed in 8-h thawed brains when compared to control and frozen wood frogs. The thaw-mediated reduction in H3K9me2 correlates well with a similarly-observed drop in H3K9me3 [6]. H3K9me2/3 are histone products of (1) ESET, a methyltransferase whose expression is reduced in brains 8 h post-freezing, and (2) G9a, a methyltransferase likely inactivated as hypoxic conditions cease. The KDM4 family (KDM4A-E), which demethylate H3K9 and are oxygen-sensitive, may also have heightened activity during the elevated O_2_ uptake/reperfusion state of thawing. The opposing trends of H3K9me2 and H3K9ac—where H3K9me2 is predominant under frozen conditions likely facilitate transcriptional repression, while H3K9ac rebounds during thawing at the expense of H3K9me2 in support of transcriptional activation, exemplifies the complexity of epigenetic crosstalk in animal extreme stress responses. 

H3K27me3 is commonly deposited at enhancer elements via the Polycomb Repressive Complex 2 (PRC2) catalytic subunit, usually Enhancer of Zeste Homolog 2 (EZH2), and is generally associated with facultative heterochromatic structures and silenced gene transcription [52]. The decreasing trend in H3K27me3 (Figure 3) during thawing, correlated with the rising trend in H3K27ac levels (Figure 2) may suggest the activation of these enhancers at 8-h thawing to stimulate transcription, possibly of constitutive genes turned off during the freeze. Larger sample sizes in future studies may indicate a thaw-mediated depletion in H3K27me3. Interestingly, elevated genomic H3K27me3 is a consequence of mouse myocardial reperfusion injury [53], and therefore, controlling H3K27me3 levels may activate genes protective from reoxygenation (like antioxidant enzymes), as the wood frog response to reperfusion is superior to mice. Finally, studies on mouse embryonic fibroblasts (mEFs) have discovered a link between DNA hypomethylation and a striking loss of broad H3K27me3 deposition [54], a link we similarly observe during wood frog brain freeze recovery [49].

## 5. Conclusions

The present study uses immunoblot analysis of isolated histone extracts to provide compelling evidence for epigenetic-mediated changes in histone proteins/PTMs during freezing and thawed recovery conditions of wood frog brains. Under frozen conditions, permissive modifications H2BK5ac, H3K4me3, and H3K14ac were significantly (*p* < 0.05) depressed while histone proteins H1.0 and H4 were significantly (*p* < 0.05) elevated. Altering trends in H3R8me2a, H3K9me2 and H3K9ac correlate with other findings and are noteworthy. Raised H3K4me3, contrasted with a significant (*p* < 0.05) drop in H3K9me2 are hallmarks of brain-thawed recovery. The current data agree with the literature, presenting evidence in support of suppressed global transcription during the MRD of whole-body freezing. Histone PTMs also point toward a rebounded transcriptional state at 8-h thawed recovery, which has been suggested previously. Strong similarities of our results on isolated histones with previous studies on nuclear/total protein samples provide validation of the histone extraction method. Overall, our findings contribute to a deeper understanding of the epigenetic and metabolic mechanisms underlying freeze tolerance in wood frogs and pave the way for future research on the intricate roles of epigenetic histone modification within animal responses to extreme environmental stress.

## Data Availability

All full immunoblot images are attached in a supplemental file (Figure S1) alongside this manuscript. The data that support the findings of this study are available from the corresponding author upon reasonable request.

## Supplementary Materials

The following supporting information can be downloaded at: https://www.mdpi.com/article/10.3390/biom14070839/s1, Figure S1: Full Western immunoblot images.
