# Supplementary material for: Reversible Histone Modifications Contribute to the Frozen and Thawed Recovery States of Wood Frog Brains
Source: Biomolecules. 2024 Jul 12;14(7):839. doi: 10.3390/biom14070839 (PMC11275241; doi:10.3390/biom14070839)

Full Western immunoblot images

Figure 1B

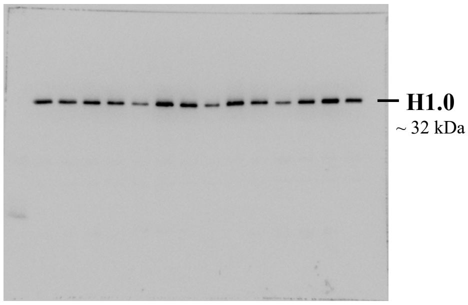

H1.0
~ 32 kDa

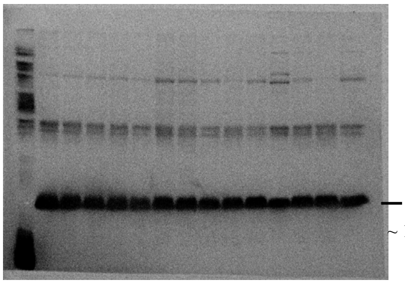

H3
~ 17 kDa

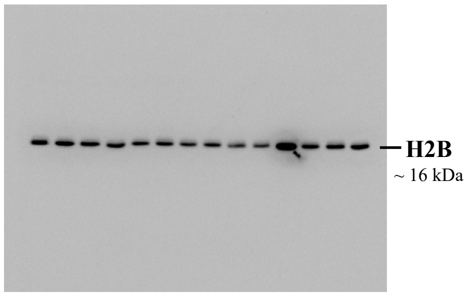

H2B
~ 16 kDa

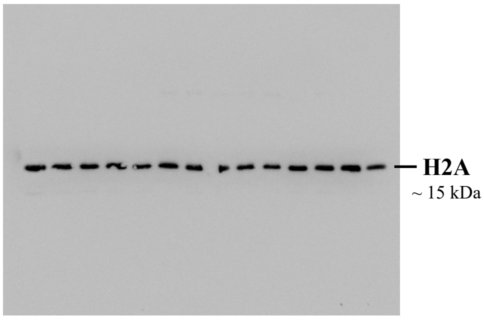

H2A
~ 15 kDa

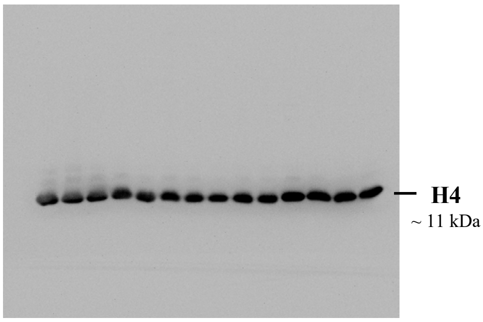

H4
~ 11 kDa

Figure 2

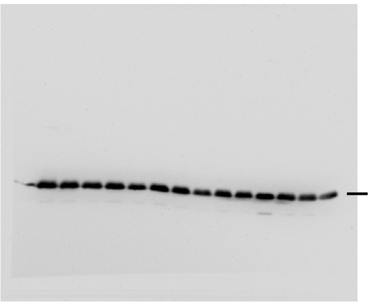

H2AK5ac
~ 15 kDa

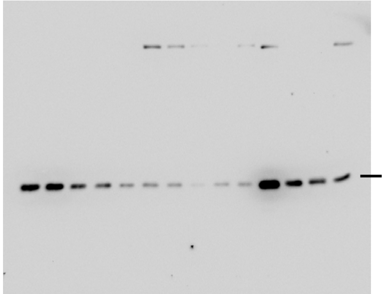

H2BK5ac
~ 16 kDa

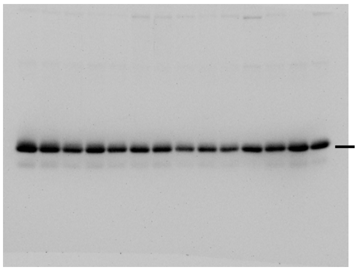

H3K9ac
~ 17 kDa

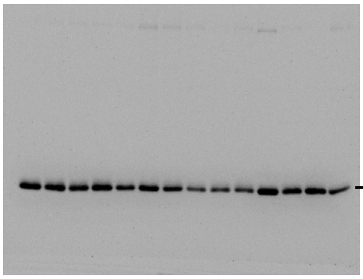

H3K14ac
~ 17 kDa

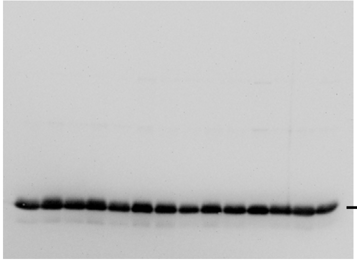

H3K18ac
~ 17 kDa

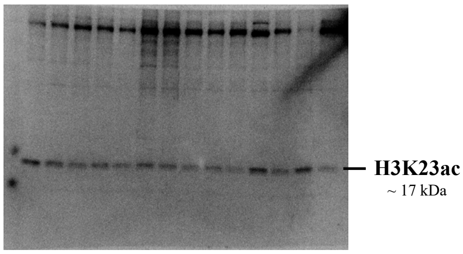

H3K23ac
~ 17 kDa

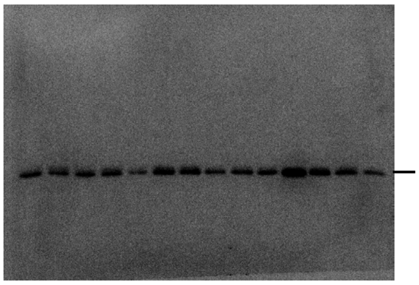

H3K27ac
~ 17 kDa

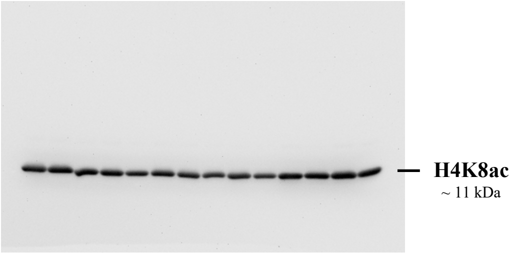

H4K8ac
~ 11 kDa

Full Western immunoblot images - continued

Figure 3

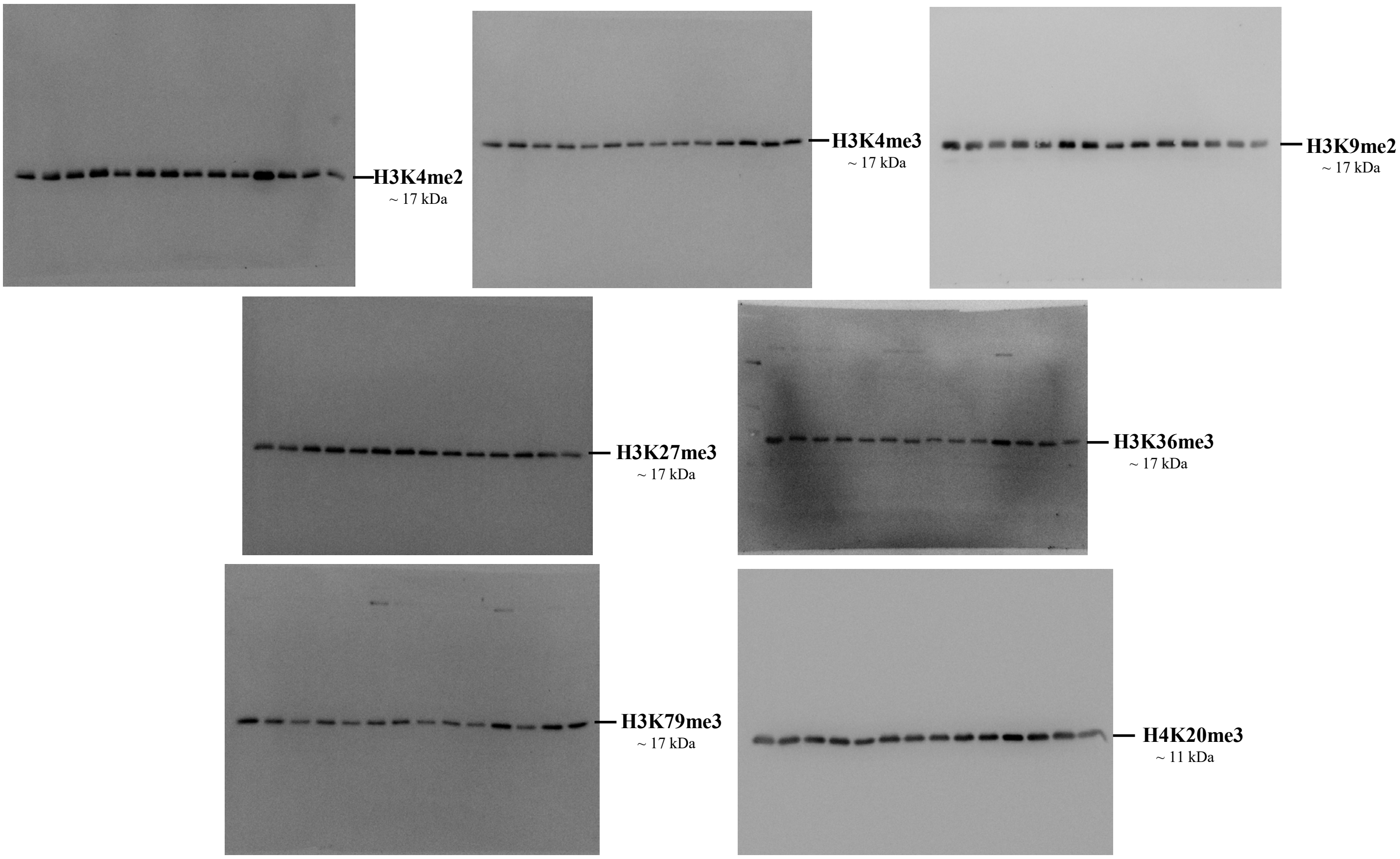

Figure 4

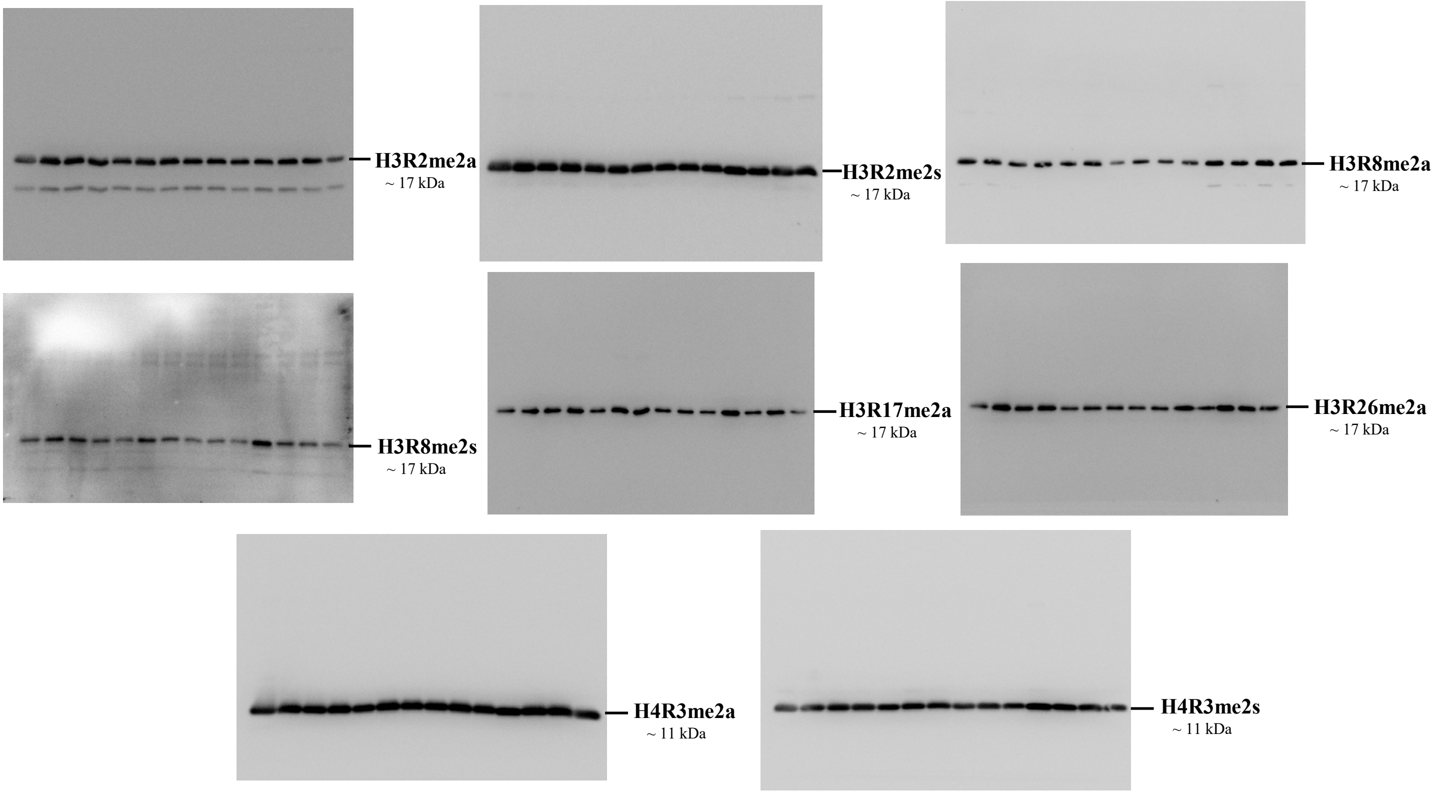

Supplement: Supplementary file 1 [file biomolecules-14-00839-s001.zip › Figure S1. Full Western immunoblot images.pdf]
